# 70th Anniversary Collection for the Microbiology Society: *Microbial Genomics*

**DOI:** 10.1099/mgen.0.000040

**Published:** 2015-12-14

**Authors:** Stephen Bentley, Nicholas Thomson

**Affiliations:** Wellcome Trust Sanger Institute, Wellcome Trust Genome Campus, Hinxton CB10 1SA, UK

## Abstract

In the context of the Microbiology Society's 70th anniversary celebration, *Microbial Genomics* (*MGen*) is the new baby of the Society's publishing family. Born on 15 July 2015, it is still in its infancy but already showing promising signs, and we have great hopes and expectations for the future. The journal captures a new and expanding area of research, one which is already having a major impact on research in microbiology, and has and continues to accelerate discoveries in the field.

*MGen* aims to publish novel and exciting science where genomic and related datasets have been applied to empower our understanding of the biology across the complete breadth of microbial life, from cycling of atmospheric gases to the molecular details of cell-to-cell interactions. We are striving to publish the best aspects of the current research from microbial genomics groups around the world, and track very closely the new innovations and revolutions that are happening at the end of every pipette and inside every computer. To do this, we have put together an Editorial Board of internationally renowned scientists with a commitment to gender-equality and support for women in STEM (Science, Technology, Engineering and Mathematics) fields. The inaugural Editorial Board members hold positions in key areas of the field with the experience, energy and foresight to emphasize the biological insights being discovered through the transformative and rapidly changing discipline that is genomics.

Genomics is the epitome of a ‘big data’ environment and, accordingly, one where the uninitiated reader can be overwhelmed and repelled in equal measure by giant lists and highly detailed visualizations. With *MGen* we focus on publishing papers that highlight the *biology* behind the data and present the findings in a *readable* and *understandable* manner, no matter how technical the study, to make these data *applicable* and *accessible* to a wider audience. This means that we will only publish papers that not only present data, but also succeed in using that data to provide biological insight.

As a new journal, *MGen* has adopted modern innovative publishing approaches such as no paper copy continuous online publishing and a pioneering open data policy. We are ever on the lookout for new ways to access and visualize the contents of the published work to ensure that readers and researchers can get the maximum value from the highly detailed analysis outputs.

As an online only journal we have the flexibility to *innovate* with content outside of the standard citable article model and one early example of this is a section entitled ‘Standing on the Shoulders of Giants’. Here, we celebrate the great scientists and great scientific papers that have created the foundations upon which microbial genomic studies are built. The section opened with a series of articles from and about Stanley Falkow, the ‘father of molecular microbial pathogenesis’, and is followed by features on giants such as Janet Thornton, Carl Woese, Brian Spratt and David Hopwood. These accounts are both inspirational, and can be ‘warts and all’, but ensure the ‘new’ is always connected to the fundamental science that underpins our field.

In the 5 months or so we have been accepting articles and at time of writing we have accepted 16 papers for publication. Papers are grouped into six categories (Microbial Evolution and Epidemiology, Microbial Communities, Microbe–Niche Interactions, Genomic Methodologies, Responses to Human Interventions, and Systems Microbiology) and the overwhelming majority of early submissions have fallen into the Microbial Evolution and Epidemiology category. This reflects the early development of the field and the area of current innovation. Researchers are feverishly mining historical archives and contemporary experimental and clinical samples, coupling them with high-throughput short-read sequencing and the latest methods in ‘omic’ and evolutionary analysis. We predict that the traffic will shift more towards translational and functional studies as the more fastidious organisms and hard-to-access samples come under the genomics spotlight and prototype experimental techniques, such as those analysing single cells, become more commonplace.

Here, we have chosen to highlight two papers that reflect current trends in submissions (Dallman *et al.*, 2015) and the potential transformative power of technology innovations (Booher *et al.*, 2015).

Dallman *et al.* (2015) describe a study focused on the Shiga-toxin-producing *Escherichia coli* (STEC) O157 : H7 previously known as enterohaemorrhagic *E. coli* O157 : H7 or to some as the ‘burger bar pathogen’, as will become clear below. It is a bacterium that has only relatively recently been fixed in our consciousness, fuelled largely by fast food incidents beginning in the 1980s and 1990s in the UK and USA. Perhaps, the most infamous of which was linked to the ‘Jack in the Box’ chain of fast food restaurants where in 1993 over 700 people contracted the disease from eating burgers. Much has changed since but STEC, now that it has been recognized, remains a serious food-borne threat to human health. Consumption of food contaminated with STEC is associated with bloody diarrhoea, but it is also able to cause the life-threatening haemolytic uraemic syndrome, essentially kidney failure, in ∼10 % of those infected. The study by Dallman *et al.* (2015) has a broad reach: it challenges previous notions of the evolutionary history of this pathogen, locally and globally, showing it has emerged as a clone much more recently than previously thought. They also link, or discount, previous biochemical-, molecular- and phage-based epidemiological typing approaches used to track it, and replace them with a phylogenetic framework, accessible to all, which is future proof: whole-genome data.

The UK has traditionally had a high incidence of STEC, and whilst the academic community and others have defined a series of different subtypes, these have been used inconsistently over time and by different countries/academic groups. In the UK we have tracked STEC by phage typing and since we could not afford to lose the link to this longitudinal surveillance data, the authors sequenced 1000 STECs, from animals and humans, collected over three decades. This allowed the complex evolutionary history of STEC to be updated: haemolytic uraemic syndrome is tightly correlated with STEC producing the *stx2a* toxin. This toxin is carried on a bacteriophage that can move between different lineages of STEC. By marking the presence of *stx2a* across the STEC phylogeny it was clear that this is a relatively recent acquisition, which may explain why this bacterium has only recently been recognized as causing severe disease. It is also evident that *stx2a* carrying STEC are well maintained within cattle herds and so present a long-term threat to health. They can even replace other less severe ‘non-*stx2a*’ STECs.

Looking across the UK and by linking genomic data with previous phage-type-based epidemiological surveillance data, the authors show that the current dominant phage type, PT 21/28, is a distinct clone that has rapidly expanded in the UK in the last 25 years and that this expansion is linked to the presence of the more severe toxins, including *stx2a*. This study unifies the tools needed to track and monitor this important disease, and provides the context with which to develop rational approaches to reducing its impact on human health.

The paper by Booher *et al.* (2015) is a prime example of knowledge progression due to rapid implementation of the latest technological advances. Indeed, during the course of this study the fledgling technology being employed underwent an upgrade which led to further improvements in data acquisition.

Booher *et al.* (2015) applied the PacBio (Pacific Biosciences) long-read sequencing technology to bacterial plant pathogen genomes with the specific intention to efficiently and accurately determine the sequences of genes encoding type 3 secretion system effector genes. In many Gram-negative bacterial pathogens, type 3 secretion systems are employed to detect and closely interact with eukaryotic cells, and then to inject effector proteins into the host cell that function in the development of that infection. In the case of plant pathogenic *Xanthomonas* spp. the effectors, known as TAL (transcription activator-like) effectors, once injected function as transcriptional activators of host plant genes and often have a direct influence on the outcome of a primary infection. An important drawback in the study of TAL effectors has been their remarkably repetitive structure that confounds assemblies made using short-read sequencing technologies. The assembly challenge is increased further by the TAL effector loci occurring in multiple copies on multiple replicons flanked by conserved sequences often associated with an abundance of transposon sequences, words which fill the nightmares of those developing assembly algorithms. The situation is exemplified by the lack of TAL sequences in available draft genome assemblies.

The target specificity of each TAL effector is determined by a central domain of tandem repeats of a 33–35 amino acid sequence. The repeats create a superhelical structure that wraps around the host DNA and affects host gene expression through base-specific interactions at effector residue 13. The identity of residue 13 and the number of repeats can be used to predict the target sequence in the host genome, providing vital information for understanding the dynamics of interactions between variations in host and pathogen sequences.

With PacBio data, Booher *et al.* (2015) identified errors in the two available finished reference sequences, that the research community rely so heavily on, and identified important sites of sequence variation including variations affecting TAL gene expression, reinforcing the concept that a reference genome is a working hypothesis. They also sequenced non-reference genomes and were able to assemble large (∼5 Mb) chromosomes into single contiguous sequences, resolve multiple phage sequences, including one case where the phage was shown to occasionally excise and form an extrachromosomal closed circular molecule. The prime target of assembling TAL genes was an all round success, although it did require development of bespoke bioinformatic tools, highlighting the pioneering nature of the work. Overall, the study captured the full repertoire of TAL genes in each genome, and demonstrated high levels of diversity within and between isolates.

By applying cutting-edge technology, Booher *et al.* (2015) have taken a significant step forward in decoding the interactions between host and pathogen – information vital to the design of strategies for controlling diseases in important crop plants that can have a devastating effect most notably in resource poor settings. Further studies of this type will inform greater understanding of host–pathogen co-evolution – important for assessing and predicting the long-terms effectiveness of intervention strategies. It is also worth noting that the simple modularity of TAL effector–DNA interactions has made them valuable in DNA editing technologies so that the employment of one cutting-edge technology is driving the development of another.

Clearly *MGen* represents a technology-driven research field–one that has rapidly expanded over the last decade and become much more equitable as technology has improved and costs have reduced. The innovations and the drive to apply them to tractable biological questions are relentless; indeed some questions have only become tractable through such innovations. This provides us with challenges as researchers and publishers: we must move quickly with the innovations, but cannot get so mesmerized by the technology that we forget the biology. We must also remember that the skills barrier for entry into this field can be high, so we must make papers readable and understandable for all, not just the specialists. We will often be faced with unprecedented levels of granularity in the data through vast numbers of samples and data points. This will require the creation of new ways to visualize and describe data, and new terminology and descriptors too. The data will bring new precision to our understanding of fundamental evolutionary processes and may even clarify concepts such as ‘species’. The data may also refute long-held wisdoms and could cause seismic shifts within established research communities, but through clear presentation of the results and their interpretation *MGen* can help keep the momentum of research discovery intact.

We would like to end on a quote from one of our ‘Giants’ Professor Stanley Falkow:

We had moved from sequencing at most a few hundred nucleotide pairs a day in 1978 to today's entire chromosomes in a morning. The good old days are now! We should not dwell on the past to be sure but we should not overlook the preface to it all because it still serves to teach us. It is the basis of current wisdom.
